# The Central Symbiosis of Molecular Biology: Molecules in Mutualism

**DOI:** 10.1007/s00239-017-9804-x

**Published:** 2017-08-07

**Authors:** Kathryn A. Lanier, Anton S. Petrov, Loren Dean Williams

**Affiliations:** School of Chemistry and Biochemistry, Georgia Institute of Technology, Atlanta, GA 30332-0400 USA

**Keywords:** Origin of life, RNA, Protein, Translation, Co-evolution, Assembly, Proficiency, Mutualism

## Abstract

As illustrated by the mitochondrion and the eukaryotic cell, little in biology makes sense except in light of mutualism. Mutualisms are persistent, intimate, and reciprocal exchanges; an organism proficient in obtaining certain benefits confers those on a partner, which reciprocates by conferring different benefits. Mutualisms (i) increase fitness, (ii) inspire robustness, (iii) are resilient and resistant to change, (iv) sponsor co-evolution, (v) foster innovation, and (vi) involve partners that are distantly related with contrasting yet complementary proficiencies. Previous to this work, mutualisms were understood to operate on levels of cells, organisms, ecosystems, and even societies and economies. Here, the concepts of mutualism are extended to molecules and are seen to apply to the relationship between RNA and protein. Polynucleotide and polypeptide are *Molecules in Mutualism*. RNA synthesizes protein in the ribosome and protein synthesizes RNA in polymerases. RNA and protein are codependent, and trade proficiencies. Protein has proficiency in folding into complex three-dimensional states, contributing enzymes, fibers, adhesives, pumps, pores, switches, and receptors. RNA has proficiency in direct molecular recognition, achieved by complementary base pairing interactions, which allow it to maintain, record, and transduce information. The large phylogenetic distance that characterizes partnerships in organismal mutualism has close analogy with large distance in chemical space between RNA and protein. The RNA backbone is anionic and self-repulsive and cannot form hydrophobic structural cores. The protein backbone is neutral and cohesive and commonly forms hydrophobic cores. Molecules in Mutualism extends beyond RNA and protein. A cell is a consortium of molecules in which nucleic acids, proteins, polysaccharides, phospholipids, and other molecules form a mutualism consortium that drives metabolism and replication. Analogies are found in systems such as stromatolites, which are large consortia of symbiotic organisms. It seems reasonable to suggest that ‘polymers in mutualism relationships’ is a useful and predictive definition of life.

## Introduction

Beyond the root of the tree of life lays the origin. During the origin of life, the onset of protein coding led to complex macromolecular structures and functions. The translation of mRNA into protein, catalyzed by the ribosome, set the path of biology that has dominated the biological earth for over 3.8 billion years.

## The Central Dogma of Molecular Biology

**Fig. 1:**
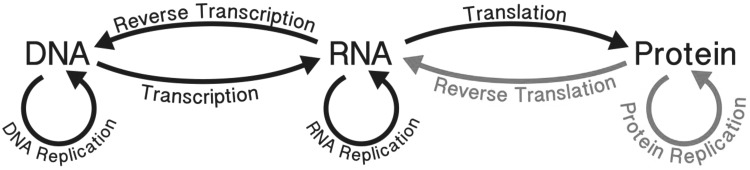
The Central Dogma describes the flow of information in biological systems. The *black arrows* are allowed processes. The *red arrows* are not observed. “Once information has got into a protein it can’t get out again” (Crick 1958) (Color figure online)

The overwhelming complexity of life rests on simple principles. Natural selection over vast time, from a common ancestor to the present, generated great diversity. The Central Dogma (Crick 1970) constrains living systems to well-defined pathways of information flow among a small number of biopolymer types (Fig. 1). Each of these linear biopolymers is formed by condensation dehydration reactions among modest sets of monomer units (Voet and Voet 2011). Biological information is represented by sequences of linked monomer units.

## Molecules in Mutualism: A Unifying Principle

The goal here is to extend important principles of biology to underlying molecules, extending the scope and explanatory power. We believe structure, function, and evolution of biopolymers are explained and best-described by their relationships with each other. *RNA and protein are Molecules in Mutualism*. This defining principle of biology and biochemistry has explanatory power comparable to the Central Dogma. Molecules in Mutualism is a rigorous and predictive definition of life.

## What is Mutualism?

**Fig. 2:**
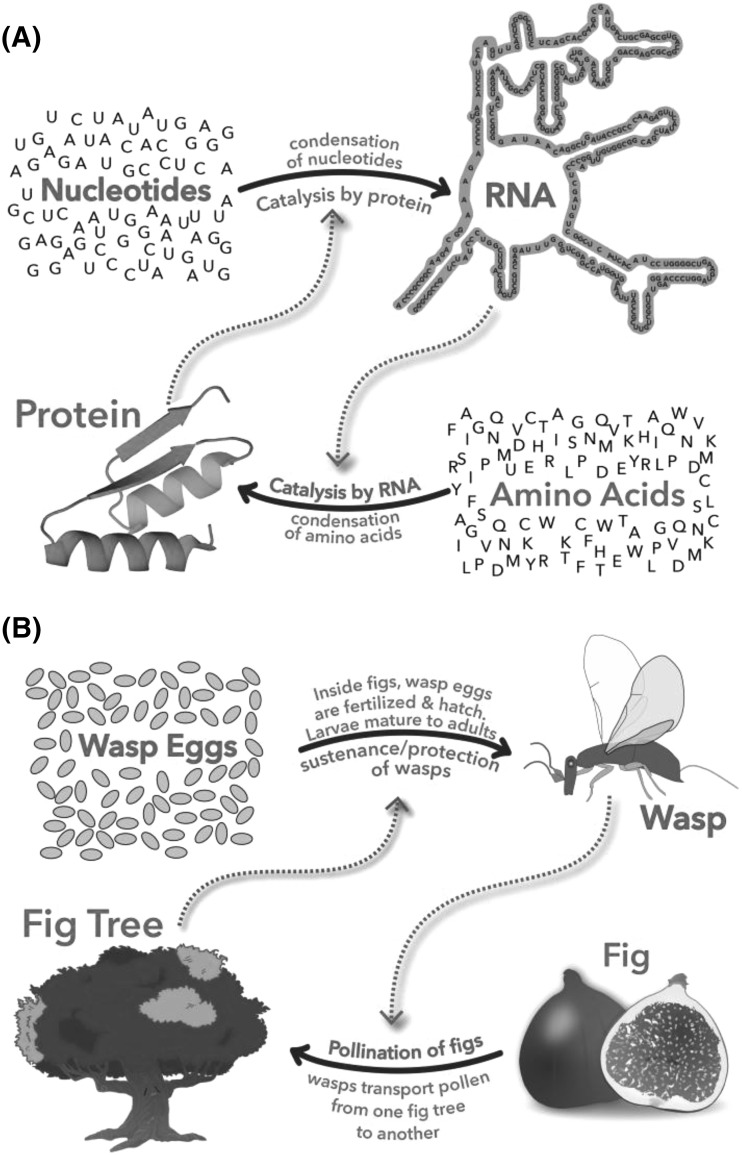
**a** Molecular mutualism. RNA makes protein. Protein makes RNA. The interdependence of RNA and protein signifies Molecules in Mutualism. **b** The fig-wasp mutualism. The fig depends on wasps to pollinate fig flowers and initiate seed production. The wasp depends on the fig for nourishment and production of offspring. Each wasp larvae consumes one would-be seed and develops within a fig fruit

A mutualism (Fig. 2) is a persistent and intimate interaction that benefits partnering species (Douglas 2015). Mutualism is reciprocal exchange; a species proficient in obtaining certain benefits confers those onto a partner, which reciprocates by conferring different benefits (Schwartz and Hoeksema 1998). Mutualisms are everywhere in the biosphere and are fundamentally important in evolution and ecology (Bronstein 2015). Mutualisms (i) sponsor co-evolution, (ii) foster innovation, (iii) increase fitness, (iv) inspire robustness, (v) are resilient and resistant to change, and (vi) involve partners that are distantly related with contrasting yet complementary proficiencies.

### Levels of Mutualism

Mutualisms are understood to operate on levels of cells, organisms, ecosystems, and even societies and economies. The eukaryotic cell is a culmination of mutualism between simpler prokaryotic cells (Sagan 1967; Poole and Gribaldo 2014; Gray 2017). The majority of land plant families are mycorrhizal; this plant-fungi mutualism is traceable to the origins of land plants (Wang and Qiu 2006). Flowering plants such as the fig (Ficus spp., Moraceae) and insects such as the fig wasp (Agaonidae, Chalcidoidea) form obligate mutual relationships (Fig. 2b) (Machado et al. 2005). The wasp depends on the fig for food and the fig depends on the wasp for pollination. Pollen-bearing female wasps initiate seed production in the fig by delivering pollen. The fig provides each wasp larva with a fig seed, which is consumed by the wasp. Essentially every species on Earth is involved in mutualisms.

### Mutualisms at the Level of Molecules

We propose that formalisms developed previously for describing mutualisms on levels of cells, organisms, and ecosystems apply equally to biopolymers. The mutual benefit, exchange of proficiencies, persistence, interdependence, co-evolution, and innovation that characterize mutualism relationships on cellular and species levels have direct parallels in the behaviors of biopolymers. Molecules in Mutualism describes, illustrates, explains, and predicts behaviors of biopolymers and provides molecular models of co-evolution, complementary structure, and co-function. Mutualism is evident in the co-synthesis of protein by RNA and RNA by protein (Fig. 2a).

## Predictions of Molecules in Mutualism

One of our goals is to use the concept of Molecules in Mutualism to help understand the properties, origins, and evolution of biopolymers. Formalisms of mutualism, when applied to biopolymers, suggest:

(i) Dependence—RNA synthesizes protein and protein synthesizes RNA (Fig. 2a),
(ii) Complementary proficiencies—structures and functions of biopolymers can be fully understood only in context. RNA explains protein and protein explains RNA,
(iii) Co-evolution—RNA and protein backbone structures and sidechains co-evolved and created each other,
(iv) Fitness—RNA and protein in combination are more fit than either alone,
(v) Innovation—neither RNA nor protein is possible or would have been achieved without the other,
(vi) Robustness—the backbone structures of RNA and protein have been fixed for billions of years, and
(vii) Resilience—RNA and protein form the original and most ancient mutualism in the biological world. Molecular mutualisms predate organismal mutualisms.

## Trading Proficiencies

### Mutualism is Exchange of Proficiencies

In mycorrhizal mutualisms, fungi are proficient in nutrient absorption, while plants synthesize and provide carbohydrates (Wang and Qiu 2006). If RNA and protein are mutualism partners, then these two polymers should also trade proficiencies. What are the specific proficiencies of each polymer type and how does their exchange benefit both partners?

### RNAs are Informational

RNA maintains, records, and transmits sequence information. Proteins are functional. Protein precisely places a broad array of active functional groups at specific positions in three-dimensional space, contributing enzymes, fibers, adhesives, pumps, pores, switches, and receptors.

### The Proficiencies of Protein and RNA Might Appear to Overlap

Proteins and RNAs both form enzyme-like entities in biological systems (Kruger et al. 1982; Guerrier-Takada et al. 1983). The ribosome, with a fully rRNA catalytic site (Ban et al. 2000), catalyzes peptidyl transfer. However, catalytic function of the ribosome requires proteins (Khaitovich et al. 1999); the ribosome is not a protein-independent ribozyme. Further, all other biological ribozymes discovered thus far only perform suicide phosphoryl transfer functions and do not turn over. Except for the ribosome, biological ribozymes do not turn over and formally are not enzymes. Although ribozymes have correctly assumed a great deal of importance in discussions of the origin of life, and do have symbolic significance, to a first approximation the great diversity of chemical reactions in biological systems are catalyzed and regulated by proteins.

## Co-evolution and Innovation

### Co-evolution of Species

Because mutualisms are prolonged and intimate, partners in mutualism influence each other’s evolution. Evolutionary change of one partner triggers change of the other. Mutualism-spawned co-evolution is illustrated in the fig-wasp mutualism (Fig. 2b). Pollen collection and deposition behaviors of fig wasps co-evolved in concert with structural adaptations in fig flowers (Machado et al. 2005).

Co-evolution increases the space available for phenotypic exploration and innovation. The symbiont-to-organelle transition that gave rise to the eukaryotic cell has led to the most profound innovations in biology (Sagan 1967; Margulis 1970; Poole and Gribaldo 2014; Gray 2017). The eukaryotic mutualism is characterized by accelerated rates of change (Brown et al. 1979), and has accomplished astounding achievements in metabolism, regulation, genetic structure, and cellular architecture.

### Co-evolution of Biopolymers

Here we explain what we mean chemically when we say RNA and protein ‘taught’ each other to assemble and function. Framed by known processes of co-evolution and innovation observed in species-level mutualisms, Molecules in Mutualism makes predictions about the evolutionary history of RNA and protein. Molecules in Mutualism supports models in which biopolymers, like the mitochondrial endosymbiont and its host cell, are products of co-evolution (Hsiao et al. 2009; Kovacs et al. 2017; Lupas and Alva 2017).

When we say, ‘proteins learned to fold,’ we mean non-coded prebiotic oligomers that were unable to fold to discrete globular structures were progressively converted to coded protein that folds spontaneously. In one reasonable scenario for this process, random sequence oligo-esters (Rich 1971; Fox and Naik 2004) which *cannot* form secondary structures, were incrementally enriched in peptide (Mamajanov et al. 2014; Forsythe et al. 2015), forming β-hairpins, then pure homochiral polypeptide, which forms β-sheets, α-helices, and tertiary and quaternary interactions (Söding and Lupas 2003; Hsiao et al. 2009; Kovacs et al. 2017; Lupas and Alva 2017). This process took place in a sea of RNA, which was also undergoing evolution. In sum, RNA and protein evolution were emergent on their co-assembly and were concurrent with evolution of the genetic code. Evidence for this model of biopolymer co-evolution is found within ribosomal structures.

## Chemical Distance

Organismal-level mutualisms are generally characterized by large phylogenetic distances, for example between metazoans and the microbes that live within their alimentary tracts. Large phylogenetic distance yields great differences in metabolic or functional proficiencies. It is less likely that two primate species, for example, would develop a mature mutualism because the partner proficiencies are similar rather than complementary.

The large phylogenetic distance in organismal-level mutualisms should have parallel in large distance in chemical and structural space in Molecules in Mutualism. Indeed, there is vast chemical and structural distance between RNA and protein. The RNA backbone is anionic and self-repulsive and cannot participate in a hydrophobic structural core. The protein backbone is neutral and cohesive and readily forms hydrophobic cores. RNA primarily uses sidechain–sidechain interactions for assembly. Protein primarily uses backbone–backbone interactions for assembly, in the formation of α-helices and β-sheets. RNA contains few types of sidechains that are all chemically similar. Protein has many types of sidechains that are chemically diverse.

## Robustness

Organismal-level mutualisms are protective and robust. Species survival is predicted in part by extent of engagement in mutualisms (James et al. 2012). This protective function helps explain the persistence of mutualisms. The mutualism that forms the basis for the eukaryotic cell is around 1.5 billion years old, while the plant-fungi mutualism is around 0.5 billion years old (Wang and Qiu 2006).

Molecular mutualisms are more persistent, and have endured for an even greater period of time than any organismal or cellular mutualism. The RNA–protein mutualism initiated before the last universal common ancestor (Woese and Fox 1977; Woese et al. 1978; Woese 2002) and has persisted for nearly 4 billion years.

## Interdependence

In organismal and cellular mutualisms, failure by either partner to provide benefit reduces the fitness of both partners. Molecules show the same intensity of interdependence. RNA makes protein in the ribosome; protein makes RNA in polymerases. Nucleoside biosynthesis consumes amino acids. Amino acid biosynthesis consumes nucleotide triphosphates.

## DNA and Other Polymers

Thus far, our discussion has focused on RNA and protein, and has excluded other polymers. In fact, on an organismal level, the number of species linked in symbiotic relationships can be large, resulting in large consortia (Orphan et al. 2002). Stromatolites contain cyanobacteria, sulfate reducers, sulfur-oxidizers, and aerobic heterotrophs that, in combination, drive the precipitation of calcium carbonate (Dupraz and Visscher 2005). By analogy with species-level consortia, one can consider a cell to be a consortium of polymers in which nucleic acids, proteins, polysaccharides, phospholipids, and other molecules form a multimember mutualism that drives metabolism, replication, transcription, and translation. It is possible that some biopolymers such as DNA originally arose as sympatric cheaters (Borges 2015) that gained advantage from a RNA–protein mutualism but did not originally contribute proficiency. Many organismal mutualisms are characterized by cheating sibling species, which can be similar to one of the mutualism partners, and which can be incorporated into the pre-existing mutualism relationship.

## A Fossil Record of the Origins of Molecular Mutualism

Over the past few years we (Hsiao et al. 2009; Fox et al. 2012; Petrov et al. 2014, 2015; Kovacs et al. 2017) and others (Söding and Lupas 2003; Bokov and Steinberg 2009; Krupkin et al. 2011; Lupas and Alva 2017) have constructed atomic level ‘movies’ of protein and rRNA evolution. These movies, based primarily on data derived from ribosomal structures, suggest incremental and hierarchical evolution of protein-type polymers in concert with incremental evolution of RNA-type polymers. During the development of the ribosome, protein “learned” to fold as RNA “learned” to base pair. Biopolymers “taught” each other to assemble and function. These anthropomorphic analogies of chemical phenomena are explained in the narrative above. In short, protein evolution was continuously guided and accelerated by interactions with rRNA. RNA evolution initiated prior to protein evolution, but after the initial steps, was guided and accelerated by interactions with protein. RNA and protein, at the very origins of biology, established a molecular mutualism that led to the Central Dogma. Molecules in Mutualism is consistent with the hypercycle model of Eigen and Schuster, which assumes cooperation within a linked assemble (Eigen and Schuster 1977).

## Summary

Lehman and coworkers (Vaidya et al. 2012; Higgs and Lehman 2015) previously argued that cooperative systems facilitated the emergence and early evolution of life. The importance of cooperative systems is an important realization. However, the constraint of their model to a single type of molecule (RNA) is inconsistent with advantages conferred by mutualisms, which involve distantly related partners with widely dissimilar proficiencies.

Little in biology makes sense except in light of *mutualism*. For example, the eukaryotic cell can be understood only in the context of mutualism relationships. Here we propose that the analogous logic and types of processes extend to biological molecules. RNA and protein created each other in the course of macromolecular co-evolution, which preceded the advent of the Central Dogma. This co-evolutionary process left imprints in the core of the ribosome, which is an ancient molecular fossil. We believe that structure, function, and origins of biopolymers (Lanier and Williams 2017) can be fully understood only in the context of their mutualism relationships.
